# Draft Genome Sequence of *Anoxybacillus* *ayderensis* Strain MT-Cab (*Firmicutes*)

**DOI:** 10.1128/genomeA.00547-17

**Published:** 2017-06-29

**Authors:** Vera Thiel, Marcus Tank, Lynn P. Tomsho, Richard Burhans, Scott E. Gay, Trinity L. Hamilton, Stephan C. Schuster, Donald A. Bryant

**Affiliations:** a Department of Biochemistry and Molecular Biology, The Pennsylvania State University, University Park, Pennsylvania, USA; b Department of Biological Sciences, Tokyo Metropolitan University, Hachioji, Tokyo, Japan; c Department of Geosciences, Penn State Astrobiology Research Center (PSARC), The Pennsylvania State University, University Park, Pennsylvania, USA; d Department of Biological Sciences, University of Cincinnati, Cincinnati, Ohio, USA; e Singapore Center for Environmental Life Sciences Engineering, Nanyang Technological University, Singapore; f Department of Chemistry and Biochemistry, Montana State University, Bozeman, Montana, USA

## Abstract

The draft genome of the Gram-positive spore-forming *Anoxybacillus ayderensis* strain MT-Cab (*Firmicutes*), isolated from an enrichment culture of *Chloracidobacterium thermophilum*, was sequenced and comprises 2,577,015 bp in 92 contigs. The draft genome is predicted to consist of 2,699 protein-coding genes, 73 tRNA-coding genes, and an estimated 8 rRNA operons.

## GENOME ANNOUNCEMENT

*Anoxybacillus ayderensis* strain MT-Cab is a thermophilic heterotrophic spore-forming member of the phylum *Firmicutes*. The strain was obtained from the original enrichment culture of *Chloracidobacterium thermophilum* isolated from a phototrophic microbial mat in an effluent channel of Octopus Spring, an alkaline siliceous hot spring in the Lower Geyser Basin of Yellowstone National Park, WY (1–3).

Strain MT-Cab was isolated in axenic culture, and its genome was sequenced to understand possible interactions among *C. thermophilum* and its chemoheterotrophic partners. *A. ayderensis* strain MT-Cab was grown in liquid LB medium; genomic DNA was extracted using the cetyltrimethylammonium bromide (CTAB) 2012 protocol of the DOE Joint Genome Institute (http://jgi.doe.gov/user-program-info/pmo-overview/protocols-sample-preparation-information/) and sequenced on an Illumina MiSeq instrument. The draft genome was assembled with Newbler (version 2.9; Roche) from 2,347,496 reads that had an average length of 301 bp. Following assembly, contigs with ≥150× coverage were maintained for genome analysis. The resulting 92 contigs comprised 2,577,015 bp, with an average G+C content of 42%.

The genome sequences of strain MT-Cab are 97.6% identical by average nucleotide identity (ANI) to the type genome of *Anoxybacillus ayderensis* (4), with 89.5% coverage of the genome. Annotation using RAST (5) predicted 2,699 protein-coding genes and 73 tRNA genes. Assembly of ribosomal rRNA genes was incomplete because of the presence of multiple copies. Based on coverage, 8 rRNA operons are predicted, which corresponds to the same number found in the *Anoxybacillus flavithermus* MK1 genome (6). Phyla-AMPHORA (7) identified all 168 *Firmicutes*-specific phylogenetic marker genes. Based on gene content, strain MT-Cab is predicted to be a spore-forming facultatively anaerobic chemoorganoheterotroph. Genes encoding enzymes for glycolysis, the tricarboxylic acid cycle, and the oxidative pentose phosphate pathway are complete. The genome lacks genes for nitrate and nitrogen reduction but has transporters for ammonia and several amino acids. Genes encoding branched-chain amino acid biosynthesis, as well as assimilatory sulfate reduction, are present in strain MT-Cab but missing in the *C. thermophilum* genome (8), indicating a possible cross-feeding in the mixed enrichment culture.

A complete respiratory electron transport chain is encoded in the genome, including an NADH dehydrogenase containing 11 subunits, succinate dehydrogenase, a menaquinol-cytochrome *c* reductase, 3 quinol oxidases (cytochrome *bd* and 2 aa_3_ type), and 2 cytochrome *c* oxidases [cytochrome *bb*_*3*_ quinol and *b*(*o/a*)_3_ types]. Genes encoding enzymes involved in mixed acid as well as lactate and butyrate, fermentation could enable anaerobic growth. The genome encodes a complete pathway for menaquinone synthesis. The presence of the sporulation-specific master regulator gene *spo0A* and RNA polymerase sigma factors *sigE, sigF*, *sigG*, and *sigK* indicates the potential for sporulation, which has been observed in culture. Similar to other *Anoxybacillus* and *Bacillus* species, strain MT-Cab is pale yellow in color due to the presence of carotenoids, putatively glycosyl-apo-8-lycopene, as identified in previous studies (9) and suggested by high-performance liquid chromatography (HPLC) analysis. The *crtBEIMN* and *Nb* genes for carotenoid biosynthesis, as well as a gene encoding a glycosyltransferase, were identified in the genome. Precursors for the synthesis of carotenoids and other isoprenoid compounds are produced by the 2-C-methyl-d-erythritol 4-phosphate/1-deoxy-d-xylulose 5-phosphate (MEP/DOXP) pathway.

### Accession number(s).

The draft genome sequence of *Anoxybacillus ayderensis* strain MT-Cab has been deposited at DDBJ/EMBL/GenBank as a whole-genome shotgun project under the accession number NDEZ00000000.
